# Supplementary material for: Conformation, Self-Organization and Thermoresponsibility of Polymethacrylate Molecular Brushes with Oligo(ethylene glycol)-block-oligo(propylene glycol) Side Chains
Source: Polymers (Basel). 2021 Aug 13;13(16):2715. doi: 10.3390/polym13162715 (PMC8400288; doi:10.3390/polym13162715)
Supplement: Supplementary file 1 [file polymers-13-02715-s001.zip › polymers-1332167-supplementary.pdf]

Supplementary Materials

Conformation, Self-Organization and Thermoresponsibility of Polymethacrylate Molecular Brushes with Oligo(Ethylene Glycol) -Block-Oligo(Propylene Glycol) Side Chains

Maria Simonova [1,*], Denis Kamorin [2,3], Oleg Kazantsev [2] , Maria Nepomnyashaya [4] and Alexander Filippov [1]

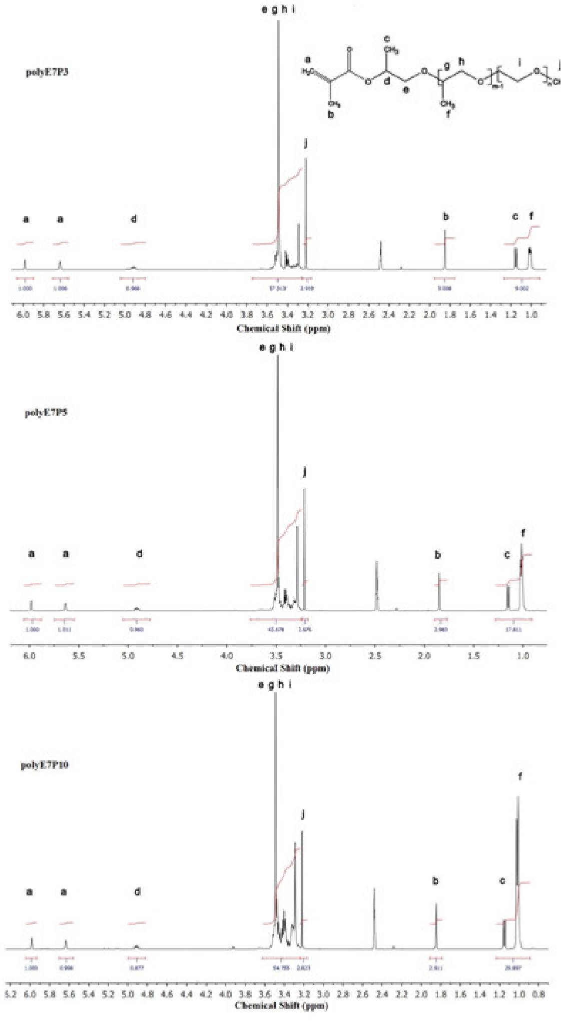

Figure S1. ^{1}H NMR spectra of the monomers in DMSO-D_6.

Molecular weight distributions of copolymers were determined by gel permeation chromatography using a Chromos LC-301 instrument with isocratic pump Alpha-10, refractometric detector Waters 410 and two exclusive columns Phenogel 5u 50A by Phenomenex, tetrahydrofuran was used as a mobile phase. Polystyrene calibration was used.

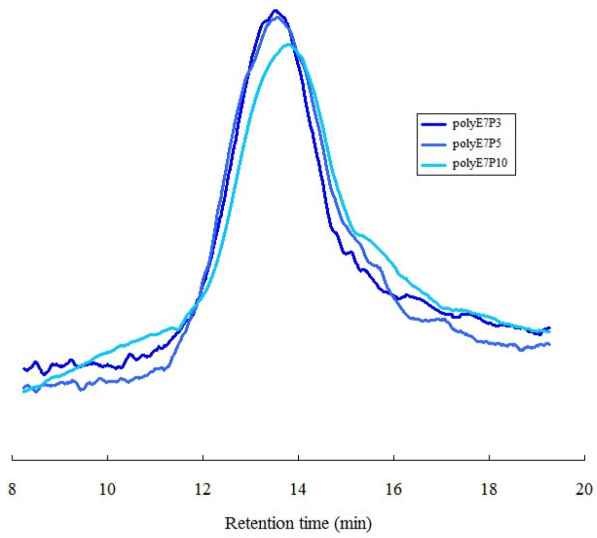

Figure S2. GPC traces of the copolymers investigated.

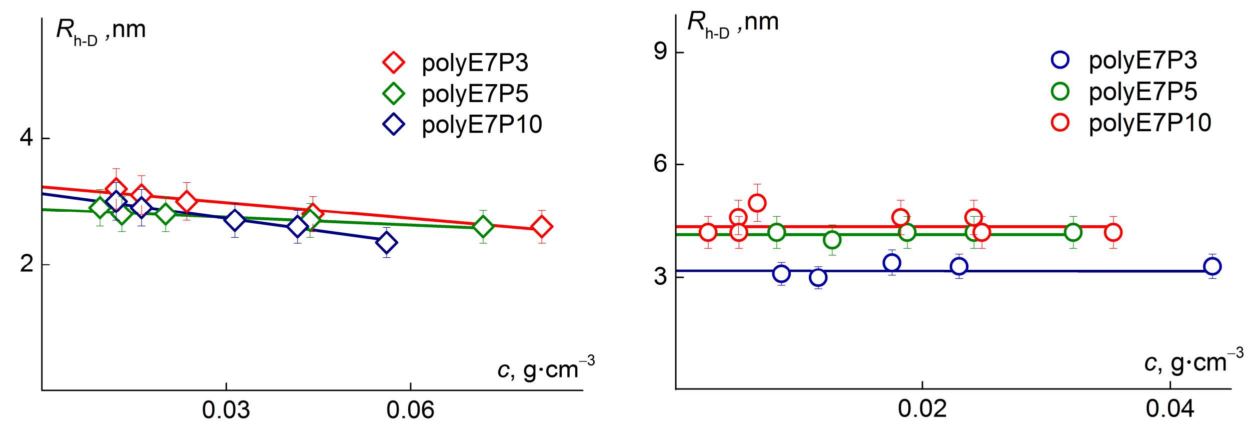

Figure S3. Concentration dependences of radius R_{h-D} for OEGeOPGpMA in acetonitrile (right) and water (left).

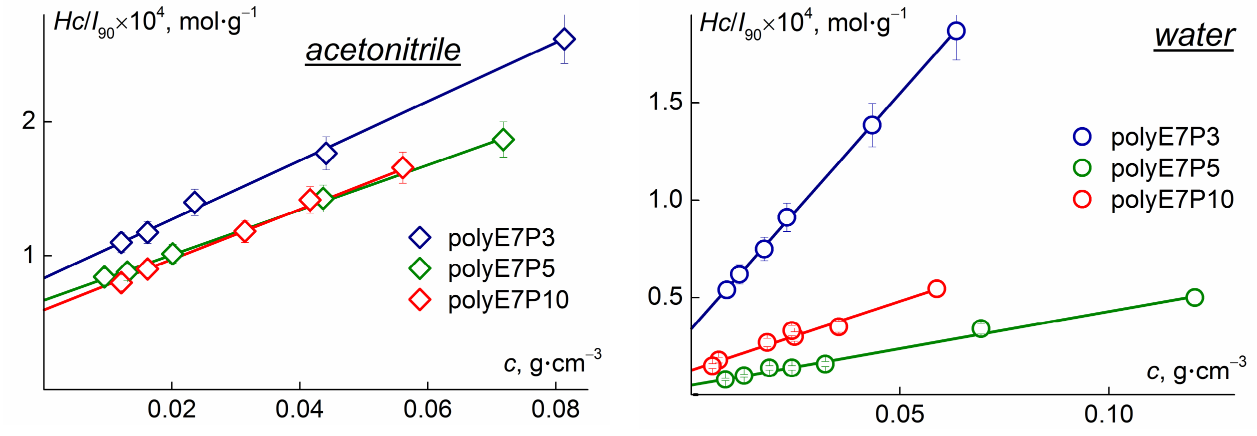

Figure S4. Debye plots for OEGeOPGpMA in acetonitrile and water.
